# The utility of chest x-ray and lung ultrasound in the management of infants and children presenting with severe pneumonia in low-and middle-income countries: A pragmatic scoping review

**DOI:** 10.7189/jogh.12.10013

**Published:** 2022-12-23

**Authors:** Saniya Kazi, Hayley Hernstadt, Yara-Natalie Abo, Hamish Graham, Megan Palmer, Stephen M Graham, ARI Review group, Trevor Duke, Hamish Graham, Steve Graham, Amy Gray, Amanda Gwee, Claire von Mollendorf, Kim Mulholland, Fiona Russell, Maeve Hume-Nixon, Saniya Kazi, Priya Kevat, Eleanor Neal, Cattram Nguyen, Alicia Quach, Rita Reyburn, Kathleen Ryan, Patrick Walker, Chris Wilkes, Poh Chua, Yasir Bin Nisar, Jonathon Simon, Wilson Were

**Affiliations:** 1 Murdoch Children’s Research Institute, Melbourne, Victoria, Australia; 2 Royal Children’s Hospital Melbourne, Melbourne, Victoria, Australia; 3 Monash Health, Melbourne, Victoria, Australia; 4 University of Melbourne Department of Paediatrics, Melbourne, Victoria, Australia; 5 Department of Pediatrics and Child Health, Faculty of Medicine and Health Sciences, Stellenbosch University, Cape Town, South Africa

## Abstract

**Background:**

Chest x-ray (CXR) is commonly used (when available) to support clinical management decisions for child pneumonia and provide a reference standard for diagnosis in research studies. However, its diagnostic and technical limitations for both purposes are well recognised. Recent evidence suggests that lung ultrasound (LUS) may have diagnostic utility in pneumonia. This systematic scoping review of research on the utility of CXR and LUS in the management of severe childhood pneumonia aims to inform pragmatic guidelines for low- and middle-income countries (LMICs) and identify gaps in knowledge.

**Methods:**

We included peer-reviewed studies published between 2000 and 2020 in infants and children aged from one month to nine years, presenting with severe pneumonia. CXR studies were limited to those from LMICs, while LUS studies included any geographic region. LUS and CXR articles were mapped into the following themes: indications, role in diagnosis, role in management, impact on outcomes, and practical considerations for LMIC settings.

**Results:**

85 articles met all eligibility criteria, including 27 CXR studies and 58 LUS studies. CXR studies were primarily observational and examined associations between radiographic abnormalities and pneumonia aetiology or outcomes. The most consistent finding was an association between CXR consolidation and risk of mortality. Difficulty obtaining quality CXR images and inter-reader variability in interpretation were commonly reported challenges. Research evaluating indications for CXR, role in management, and impact on patient outcomes was very limited. LUS studies primarily focused on diagnostic accuracy. LUS had higher sensitivity for identification of consolidation than CXR. There are gaps in knowledge regarding diagnostic criteria, as well as the practical utility of LUS in the diagnosis and management of pneumonia. Most LUS studies were conducted in HIC settings with experienced operators; however, small feasibility studies indicate that good inter-operator reliability may be achieved by training of novice clinicians in LMIC settings.

**Conclusions:**

The available evidence does not support the routine use of CXR or LUS as essential tools in the diagnosis and initial management of severe pneumonia. Further evaluation is required to determine the clinical utility and feasibility of both imaging modalities in low-resource settings.

Pneumonia is a leading cause of death in under-five children living in under-resourced settings [1]. Accurate and early diagnosis is important in reducing pneumonia-related mortality. Clinical features are non-specific, especially in young children, and there is no single test with high specificity or sensitivity for the diagnosis of pneumonia. Computed tomography (CT) of the chest may be considered a radiologic gold standard in diagnosing pneumonia, but cost, accessibility and radiation exposure make it unfeasible for routine use [2].

Currently, chest x-ray (CXR) is the primary imaging modality for pneumonia and is commonly used as a reference standard for the diagnosis of pneumonia [3]. However, some diagnostic and technical limitations of CXR have been recognised [3,4], and it is estimated that approximately half of the world’s population does not have access to imaging facilities [4]. Moreover, maintenance of radiographic equipment can be costly, and a consistent power supply and experienced personnel are needed for obtaining and interpreting images. The World Health Organization Integrated Child Handbook defines pneumonia in children aged 2-59 months as cough, and/or difficulty breathing, tachypnoea or chest indrawing, while severe pneumonia additionally includes one or more general danger signs [5]. Imaging is not currently included as a necessary component in the initial management of pneumonia, but CXR is suggested for children not responding as expected to medical management. International guidelines consistently advise against routine use of CXR for non-severe pneumonia, but recommendations for severe pneumonia vary widely [6-9].

Lung ultrasound (LUS) has been used to recognise complications of pneumonia, such as pleural effusion [10], and is increasingly being evaluated as an imaging tool in diagnosis and management of pneumonia in children [11,12]. Ultrasound is radiation-free, portable, inexpensive, and can be performed by a trained clinician at the bedside, with immediate results. These properties make it an attractive tool for resource-limited settings.

This scoping review aimed to systematically map and examine the literature on utility of CXR and LUS in children with severe pneumonia, to guide pragmatic recommendations for resource-limited settings.

## METHODS

This study was conducted following the Preferred Reporting Items for Systematic Reviews and Meta-Analyses statement for Scoping Reviews (PRISMA-ScR).

### Study selection and eligibility criteria

The search strategy was drafted by an experienced research librarian using Medical Subject Heading (MeSH) terms and keywords and refined through team discussion. We searched MEDLINE, Embase, PubMed, and Global Index Medicus for English-language studies published from January 1, 2000, to July 31, 2020. We reviewed reference lists of included papers for additional articles which may have been missed. We included studies of children (aged one month to nine years) with acute severe pneumonia which evaluated the role of either LUS or CXR in relation to one or more of our pre-defined themes: indications for the imaging, role of imaging in diagnosis (including diagnostic accuracy of LUS), role of imaging in management, impact of imaging on patient outcomes, feasibility, and practical considerations for low- and middle-income countries (LMIC). As the use of LUS for pneumonia is novel and not established globally, we included LUS studies from any geographic region, while CXR studies were limited to those from LMIC. The search strategy and details of the selection process are presented in Appendix S1 and Appendix S2 in the **Online Supplementary Document**.

### Data extraction and synthesis

We used the Covidence electronic platform (Covidence, Melbourne) for record management from screening to data extraction stages. Two reviewers independently assessed articles for relevance, and discrepancies were resolved by discussion, including a third reviewer where necessary. We used a pre-defined, custom-created data extraction template in Covidence to capture data on study design, aims, geographic setting, key findings, and limitations. Diagnostic accuracy of LUS has been explored in several recent systematic reviews and meta-analyses. Hence, we utilised the existing reviews to pool these results, so limited data were extracted from original studies evaluating LUS diagnostic accuracy. We classified research articles first by imaging modality, and then by theme. A single article could be classified into multiple thematic categories if it had more than one major focus. We utilised descriptive statistics to summarise characteristics of research papers and presented key results in a narrative format.

## RESULTS

**Figure 1:**
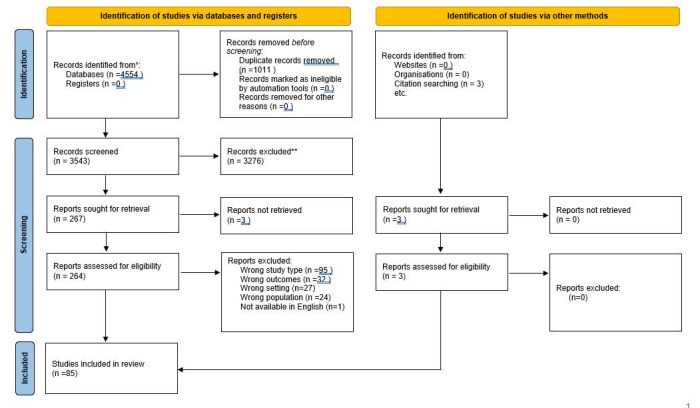
PRISMA 2020 flow diagram of literature search.

**Figure 2:**
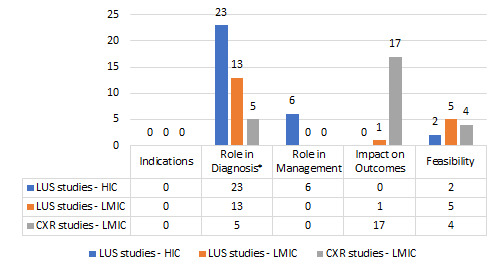
Themes of original studies. LUS – lung ultrasound, CXR – chest x-ray, HIC – high income country, LMIC – low- and middle-income countries. *21/23 LUS HIC studies and 10/13 LUS LMIC studies in this category were diagnostic accuracy studies.

Our search yielded 3540 individual studies; additional three studies were identified from references of other articles (**Figure 1**). 85 studies ultimately met our inclusion criteria: 26 original studies and one systematic review primarily evaluating CXR, and 50 original studies and eight systematic reviews related to LUS (**Figure 2**).

### Overview of studies

Of 26 CXR studies with original data, 17 (65%) included children with WHO severe or very severe pneumonia (as per the 2005 definition) while the remaining had a broader inclusion criterion for pneumonia and severity. No studies evaluated children based on the WHO 2014 definition of severe pneumonia. Most CXR studies (n = 17, 65%) provided observational data about associations between CXR abnormality and clinical course, while one systematic review addressed predictors of pneumonia severity.

Of the 58 articles examining the role of LUS in severe pneumonia, most original studies (n = 31/50, 62%) and all systematic reviews (n = 8) primarily evaluated diagnostic accuracy. One-third (n = 19, 38%) of LUS studies were conducted in LMICs, primarily addressing diagnostic accuracy (n = 10/19, 53%) and feasibility (n = 5/19, 26%).

There were no studies evaluating indications for CXR or LUS. Included studies that did examine patient outcomes examined the association between imaging findings and patient outcomes rather than the impact of using the particular imaging modality. Further details of the nature and distribution of CXR and LUS studies can be found in Appendix S3 and S4 in the **Online Supplementary Document**.

### Utility of chest x-ray in severe pneumonia

The 26 original CXR studies had heterogenous study aims and designs (**Table 1****)**. Most studies (80%) were conducted in LMICs. Only three studies were conducted solely in a low-income country (two in The Gambia and one in Ethiopia) and two were conducted over multiple sites which included low-income countries. Most CXR studies (92%) were based in emergency departments (EDs) or inpatient settings. One study was conducted in four peri-urban, primary health facility level study sites in Pakistan [18], while a large Gambian study [26] recruited children presenting to either a secondary care facility or hospital. Older children (5-9 years) were under-represented with only three studies including this age group [19,22,39].

**Table 1:** Characteristics of chest x-ray studies

Study	Country of study	Pneumonia definition and CXR criteria (if any) for inclusion	Study design	Participants	Aim(s) of study
**Role of CXR in diagnosis**					
Ferrero, 2010 [13]	Argentina, Brazil, Dominican Republic	WHO severe or very severe pneumonia (2005) and abnormal CXR	Prospective, multi-centre observational study	3-59 mo of age, N = 2536	Correlate CXR findings with pneumococcal isolation in children with severe pneumonia (from pleural effusion or bacteraemia)
Gowraiah, 2014 [14]	India	WHO pneumonia (2005) [15]	Prospective, multi-centre observational study	2-59 mo of age, N = 524	Develop clinical criteria for low-resource settings, to differentiate pneumonia from wheezy diseases and allow early detection of children at high risk of death
Nascimento-Carvalho, 2015 [16]	Brazil	Respiratory complaints and fever or difficulty breathing and detection of pulmonary infiltrate and/or pleural effusion on CXR	Cross-sectional	0-59 mo of age, N = 209	Assess if there is an association between a specific aetiology and radiologically confirmed pneumonia
Kut, 2013 [17]	Gambia	WHO severe or very severe pneumonia (2005)	Prospective observational	2-59 mo of age, N = 420	Determine factors that predict hypoxaemia at presentation in children with severe pneumonia.
Nizami, 2005 [18]	Pakistan	WHO severe or very severe pneumonia (2005)	Prospective cohort	2-59 mo of age, N = 1203, 823 had CXR	Assess the utility of CXR in management of acute respiratory infection at community level
**CXR and outcomes**					
Araya, 2016 [19]	Paraguay	Presence of respiratory signs and symptoms and history of fever and consolidation on CXR at admission	Retrospective cohort	<15 y of age, N = 860	Assess the use of a modification of PIRO scale (used in adult pneumonia) and the association between modified PIRO score and mortality, stratified by risk.
Awasthi, 2020 [20]	India	WHO pneumonia (2014) [5]	Multi-site prospective surveillance	2-59 mo of age. N = 3214, 2829 with CXR obtained and interpretable	Assess the radiological abnormalities in CXRs and identify demographic and clinical correlates of specific radiological abnormalities in children with pneumonia aged 2-59 mo
Basnet, 2015 [21]	Nepal	WHO severe or very severe pneumonia (2005) [15]	RCT*	2-35 mo of age, N = 598	Assess cohort of Nepalese children given standard pneumonia treatment, and the correlation between clinical and radiological and other variables.
Bharti, 2008 [22]	India	WHO severe or very severe pneumonia (2005) [15]	Prospective observational	<10 y of age, N = 83	Radiological appraisal in children with clinically diagnosed severe pneumonia and its association with clinical outcome
Dean, 2018 [23]	Multiple	Studies evaluating children with pneumonia, excluding those not requiring focality on exam or on CXR, or those with high proportion of wheeze	Systematic review	<18 y of age, 56 studies	Systematic review of evidence for predictors of pneumonia severity in children and risk stratification of children with pneumonia
Dembele, 2019 [24]	Philippines	WHO severe or very severe pneumonia (2005) [15]	Prospective, multi-centre case series study	0-59 mo of age, N = 5054	Define aetiological, demographic, and environmental factors, and clinical findings associated with mortality due to childhood pneumonia among hospitalised children in the Philippines
Djelantik, 2003 [25]	Indonesia	WHO severe or very severe pneumonia (2005)	Retrospective cohort	0-24 mo of age, N = 4351	Identify useful screening criteria for mortality among children hospitalised for pneumonia (developing country setting)
Enwere, 2007 [26]	Gambia	WHO severe or very severe pneumonia (2005)	Randomised control trial	0-29 mo of age, N = 5842 (9109 pneumonia episodes)	Assess incidence of different types of radiological pneumonia (primary endpoint pneumonia, other infiltrates/abnormalities pneumonia, normal CXR) and compare clinical and laboratory features between these groups (data from pneumococcal vaccine RCT)
Fancourt, 2017 [27]	Bangladesh, the Gambia, Kenya, Mali, South Africa, Thailand, Zambia	WHO severe or very severe pneumonia (2005)	Multi-country case-control study	1-59 mo of age, N = 4242, 3587 with CXR obtained and interpretable	Describe CXR findings of clinically diagnosed pneumonia cases and determine if there were differences by geography, epidemiological setting, particular clinical signs, or pneumonia risk factors
Jakhar, 2018 [28]	India	WHO severe or very severe pneumonia (2005)	Prospective cohort	Two months to five years of age, N = 120	Determine the aetiology of severe pneumonia in under-five children and study the risk factors of poor outcomes (persistence of features of severe pneumonia after 72 h or worsening of clinical condition, need for change of antibiotics, need for mechanical ventilation, prolonged hospitalization >5 d and death).
Jeena, 2007 [29]	South Africa	WHO severe or very severe pneumonia (2005)	Nested, prospected observational study	3-59 mo of of age, N = 425	Compare radiological features and outcome of WHO defined severe pneumonia among HIV infected and exposed uninfected children randomised to receive penicillin or oral amoxicillin
Kelly, 2016 [30]	Botswana	WHO pneumonia (2005)	Prospective cohort study	1-23 mo of age, N = 249	Assess if findings on CXR associated with treatment failure, need for respiratory support, length of stay, and in hospital mortality for children with pneumonia.
Kin Key, 2009 [31]	Brazil	WHO severe or very severe pneumonia (2005) + unilateral pulmonary infiltrate or pleural effusion on CXR	Prospective observational	0-59 mo of age, N = 113	Assess if chest radiograph findings present on admission are associated with severity of childhood community acquired pneumonia
Lupisan, 2007 [32]	Philippines	WHO severe or very severe pneumonia (2005)	Prospective observational	2-59 mo of age, N = 1259	Identify demographic and nutritional risk factors in infants and young children with severe pneumonia that predict death, to develop a triage system to identify severely ill children with pneumonia
Nguyen, 2020 [33]	Vietnam	WHO pneumonia (2014)	Prospective observational	2-59 mo of age, N = 3817, 2199 met WHO pneumonia case definition	Identify predictors of unlikely bacterial pneumonia among children who present to hospital with respiratory symptoms. To also identify predictors of an adverse pneumonia outcome and combine this with predictive models an existing guidance to suggest a potential pragmatic algorithm to reduce unnecessary antibiotic use and hospitalization.
Patel, 2008 [34]	Colombia, South Africa, Vietnam, Pakistan, Ghana, Mexico, India, Zambia	WHO severe or very severe pneumonia (2005)	Nested, prospective observational study	3-59 mo of age, N = 1121	Evaluated the use of CXR in predicting treatment failure in children with severe pneumonia recruited in the Amoxicillin Penicillin Pneumonia International Study (APPIS).
Seear, 2016 [35]	India	WHO severe or very severe pneumonia (2005)	Prospective cohort	0-59 mo of age, N = 134	Test the predictive accuracy and reporting reproducibility of digital chest radiographs under low resource conditions
Waris, 2016 [36]	Pakistan	Severe pneumonia, based on a clinical scoring system	Cross-sectional	2-36 mo of age, N = 581	Assess the association of haematological and radiological findings with clinical outcome in hospitalized children with severe pneumonia.
**Practical considerations**					
Bada, 2007 [37]	Peru	Acute lower respiratory tract infection and concurrent wheezing	Prospective consecutive case study	2-24 mo of age, N = 200	Assess inter-observer agreement in CXR interpretation in children with acute respiratory infection and wheezing
Correia, 2011 [38]	Brazil	Acute lower respiratory tract infection	Retrospective cohort	<5 y of age, N = 48	Assess inter-observer agreement in the interpretation of chest radiographs of children admitted with suspected pneumonia
Hassen, 2019 [39]	Ethiopia	WHO severe pneumonia (2005)	Prospective short cohort	3 mo to 14 y of age, N = 122	Assess role of CXR for diagnosis of pneumonia and association of clinical characteristics with radiologic findings, and predictors of hospitalization. Regression model done for predictors
Patel, 2007 [40]	India	WHO severe or very severe pneumonia (2005)	Randomised control trial*	3-59 mo of age, N = 172	Estimate inter-observer agreement in interpretation of chest radiographs in children with severe pneumonia, and to examine wither WHO training intervention increases the inter-observer agreement

CXR – chest x-ray, WHO – World Health Organization, RCT – randomised controlled trial, HIV – human immunodeficiency virus, mo – months, y – years

### Role of chest x-ray in diagnosis

Five studies [13,14,16-18] explored the role of CXR in diagnosis. Clinical and radiographic definitions of pneumonia varied significantly across studies. The only standardised format for interpretation of CXRs was in the use of the WHO Primary Endpoint Pneumonia criteria [3], which was common in clinical studies.

Overall, severity as per WHO-defined pneumonia (2005) correlated poorly with radiographic pneumonia diagnosis [14,18,27]. In a multi-centre study from India (n = 524), auscultation was found to be superior to CXR in distinguishing pneumonia from other respiratory disorders, using a final paediatrician diagnosis as a reference standard [14]. Nizami et al. [18] evaluated the utility of performing CXR in children presenting with fever and cough in four peri-urban, primary level health facilities in Pakistan; approximately half of children with clinical upper respiratory tract infections had radiographic opacity or infiltration considered to be suggestive of infection.

A normal CXR was commonly reported (24%-72%) in children with WHO-defined severe pneumonia [20,27,30,32,36]. The Pneumonia Etiology Research for Child Health (PERCH) study [27] evaluated the likelihood that a child with WHO-defined severe pneumonia (2005) and a normal CXR truly has pneumonia, based on the presence of two or more additional pneumonia signs, and reported that pneumonia was the “likely diagnosis” in 52% of these children. Infants (<12 months age) had the highest proportion of normal CXRs, possibly due to this age group having more cases of bronchiolitis.

Two studies [13,16], including a multi-centre study from South America (n = 2536), evaluated CXR in identifying bacterial pneumonia. Children with WHO severe pneumonia (2005) and *Streptococcus pneumoniae* isolated on blood culture or PCR were unlikely to have a normal CXR (negative predictive value (NPV) = 92%) [13]. Alveolar opacities and pleural effusion were associated with pneumococcal isolation in blood or pleural culture *(P <* .001). A study of 209 children in Brazil identified associations between a normal CXR and presumed viral pneumonia, as well as abnormal CXR and presumed bacterial pneumonia; however, assigning bacterial or viral aetiology on CXR alone would misclassify 30%-50% of cases [16].

### Chest x-ray and outcomes

Eighteen studies evaluated associations between CXR abnormalities and severity of pneumonia (**Table 2**). Five studies from countries in Africa and Asia comprising 20 754 children with severe pneumonia identified that radiographic dense consolidation at presentation was associated with a higher case fatality rate within one month of admission [24-27,32]. In two of these studies, the strength of association decreased after multivariate analysis [24,25]. The PERCH study found that radiographs of children who died were twice as likely to have WHO Primary Endpoint Consolidation (50% vs 24%) and less likely to have “other infiltrates” (18% vs 28%). A systematic review (which also included studies from high-income countries (HICs)) evaluated predictors of pneumonia severity and found multi-lobar infiltrates and moderate or large pleural effusions to be predictive of mortality, along with several other factors such as younger age and hypoxaemia [23]. A retrospective study [19] from Paraguay evaluated a multi-dimensional prognostic score for predicting pneumonia mortality; CXR abnormalities, particularly multi-lobar opacities, added value to the prediction score (odds ratio = 4.9; 95% confidence interval (CI) = 2.7-8.9).

**Table 2:** Association between chest x-ray abnormalities and outcome

			Study details	Associations between radiographic feature and outcomes				
**Study**		**Study design and participants (n)**	**CXR abnormality**	**Outcome: mortality**	**Outcome: treatment failure**	**Outcome: delayed recovery**	**Outcome: severe features**	**Study findings**
Araya, 2016 [19]	Retrospective cohort study, N = 860		Multi lobar consolidation, pleural effusion, pneumothorax	X				Multi-lobar CXR involvement (OR = 4.9; 95% CI = 2.7- 8.9; *P* < 0.0001), pleural empyema (OR = 2.6; 95% CI = 1.1-6.2; *P* < 0.05), pneumothorax (OR = 15; 95% CI = 2.9-76.6, *P* < 0.01) associated with mortality
Awasthi, 2020 [20]	Multi-site prospective surveillance study, N = 3214		Abnormal CXR				X	“Abnormal CXR” associated with fever, pallor, malnutrition, wheezing on auscultation
Basnet, 2015 [21]	RCT, N = 598		Primary Endpoint Consolidation*		X	X		Primary endpoint consolidation associated with prolonged recovery, treatment failure (as well as younger age and hypoxia)
Bharti, 2008 [22]	Prospective observational study, N = 83		Lobar consolidation		-	X		Lobar consolidation associated with longer hospital stay; no difference in time to defervescence of fever and tachypnoea between CXR findings (primary outcome)
Dembele, 2019 [24]	Prospective, multi-centre, case series, N = 5054		Consolidation, pleural effusion	X				Consolidation or pleural effusion associated with increased mortality risk (CFR all children 4.7% v 13% among consolidation group) however univariate analysis did not confirm independent association
Djelantik, 2003 [25]	Retrospective cohort study, N = 4351		Alveolar infiltrate	X				Alveolar infiltrate predictive of death (RR = 1.6, 95% CI = 1.1-2.1) however did not contribute to prediction model on multiple regression analysis
Enwere, 2007 [26]	RCT, N = 5842		PEP*	X	X		X	PEP associated with longer duration of illness, invasive bacterial disease, and higher mortality within 28 d (4% vs 0.7% with other infiltrates and 1.2% normal CXR). Children with PEP on CXR described to be “more unwell, more malnourished, more anaemic” than those with other radiographic findings. PCV vaccinated children were significantly less likely to have PEP on CXR, but clinical presentation of PEP did not differ in PCV vaccinated vs unvaccinated children.
Fancourt, 2017 [27]	Multi-country case-control study, N = 4242		Consolidation	X				CXR consolidation was associated with a higher case fatality ratio at 30-d follow-up (13.5%) compared to other infiltrates (4.7%) or normal (4.9%) CXRs
Hassen, 2019 [39]	Prospective short cohort study, N = 122		Abnormal CXR			-		No association between CXR abnormality and length of stay
Jakhar, 2018 [28]	Prospective cohort study, N = 120		Abnormal CXR		X	X		“Abnormal CXR” associated with delayed recovery (*P* < 0.001) and treatment failure (*P* < 0.001, OR = 8.45. 95% CI = 3.56-20.04), change in antibiotics (<0.001, OR = 9.66, 95% CI = 2.62-35.53))
Jeena, 2007 [29]	Nested, prospective cohort study, N = 425		Infiltrates, non-segmental consolidation		X			Other consolidation/infiltrates associated with treatment failure in HIV infected children receiving either penicillin or amoxycillin
Kelly, 2016 [30]	Prospective cohort, N = 249		PEP*		X	X		PEP independently associated with treatment failure, longer length of stay, and longer duration of respiratory support, (Insufficient deaths to assess association with mortality)
KinKey, 2009 [31]	Prospective observational study, N = 113		Upper lobe involvement				X	Upper lobe involvement on CXR and severity of pneumonia (based on WHO classification)
Lupisan, 2007 [32]	Prospective observational study, N = 1259		Dense infiltrates	X				Dense infiltrates in infants aged two to five months was independently associated with death.
Nguyen, 2020 [33]	Prospective observational study, N = 2199		Consolidation				X	Consolidation on CXR predictive of adverse pneumonia outcome
Patel, 2008 [34]	Nested, prospective observational study, N = 1121		“Significant pathology”		X			“Significant pathology” on CXR which included PEP, other infiltrates, pleural fluid was associated with treatment failure
Seear, 2016 [35]	Prospective cohort study, N = 134		Pleural effusion				X	Pleural effusion associated with more severe pneumonia (PPV 100%), however NPV low (41%)
Waris, 2016 [36]	Cross-sectional study, N = 581		Lobar consolidation		X			Lobal consolidation (OR = 6.00) associated with delayed clinical response. More than half of children had normal CXRs, including 48.8% of children with delayed response.

CXR – chest x-ray, OR – odds ratio, PEP – primary endpoint pneumonia, PCV – pneumococcal vaccine, RR – risk ratio, CI – confidence interval, RCT – randomised controlled trial, WHO – World Health Organization, PPV – positive predictive value, NPV- negative predictive value

*Primary endpoint consolidation and primary endpoint pneumonia are definitions developed by WHO for interpretation of CXR in pneumonia epidemiological studies [41]. Primary endpoint consolidation is defined as a dense consolidation or confluent opacity that occupies a portion or whole of a lobe or the entire lung, that may or may not contain air bronchograms. Primary endpoint pneumonia refers to the presence primary endpoint consolidation or pleural effusion.

### Feasibility and practical considerations for LMIC settings

Six studies reported inter-observer reliability in CXR interpretation for pneumonia. Reliability was impacted by observer experience and the radiographic finding in question [30,35,37-40] (n = 925, kappa value (ĸ) range = 0.15-0.7). Agreement was best for reporting lobar consolidation or primary endpoint pneumonia using the WHO standardised criteria for interpretation and became less reliable with more obscure findings such as “other infiltrates” [30,39]. When CXR’s with clear lobar pneumonia and pleural effusion were excluded, Bada et al. [37] identified poor concordance between junior doctors in interpreting other CXR abnormalities in children presenting with respiratory symptoms (ĸ = 0.2). An Ethiopian study found low concordance in interpretation of CXR for paediatric pneumonia among two senior radiologists when using a standardised checklist (ĸ = 0.45 for consolidation; ĸ = 0.65 for pleural effusion) [13]. Patel et al. found an improvement in agreement and reduction in diagnosis of significant pathology following training in CXR interpretation using the WHO standardised criteria [34]. Several studies from LMIC settings described significant issues with film quality, interpretation, and inter-observer variability, impacting the utility of CXR in these settings [14,20,27]. Between 4%-20% of radiographs were determined to be uninterpretable in two recent, multi-site studies from hospital settings, despite technical assistance to optimise quality [20,27]. In a study conducted at health facility level in Pakistan [18], 19% of radiographs were considered uninterpretable by the attending physician.

### Utility of LUS in Severe Pneumonia

#### Diagnostic accuracy of lung ultrasound

31 original studies and eight systematic reviews [11,42-48] evaluated diagnostic accuracy of LUS (**Table 3** and **Table 4**).

**Table 3:** Systematic reviews evaluating diagnostic accuracy of lung ultrasound

Study	Total number of studies (LMIC studies)*†‡	Inclusion criteria of studies	Pneumonia reference standard for inclusion in review	Pooled sensitivity (%)	Pooled specificity (%)
Balk, 2018 [43]	12 (2)	<18 y with suspected CAP	Any (clinical or radiological)	95.5	95.3
Heuvelings, 2019 [44]	19 (3)	<18 y with pulmonary diseases including CAP	Any imaging modality with or without clinical assessment	95	96.1
Najgrodzka, 2019 [45]	22 (1)	<18 y with suspected CAP	Any (clinical or radiological)	93.2	89.6
Orso, 2018 [46]	17 (3)	<18 y with suspected CAP	Any (clinical or radiological)	94	94
Pereda, 2015 [11]	8 (0)	<18 y with suspected CAP and confirmation by CXR or CT chest	Any (clinical or radiological)	96	93
Tsou, 2019 [42]	25 (4)	<21 y with suspected CAP	Any (clinical or radiological)	94	92
Wang, 2019 [47]	6 (0)	<18 y with suspected CAP	Expert paediatrician clinical diagnosis	96.7	87
Xin. 2018 [48]	8 (0)	<18 y with suspected CAP	Combination of clinical data, laboratory results, and imaging (CXR or CT)	93	96

LMIC – low and middle-income country, CAP – community-acquired pneumonia, CT – computerized tomography, CXR – chest x-ray, y – years

*Includes both paediatric and neonatal studies.

†There is considerable overlap between systematic reviews of the studies included in each review.

‡There were 5 original diagnostic accuracy studies from LMIC settings in total.

**Table 4:** Original lung ultrasound diagnostic accuracy studies

Study	Study design	Country	Inclusion criteria	Participants (n)	Reference standard for pneumonia
**Low- and middle-income country**					
Ahmad, 2019 [49]	Prospective observational study	Pakistan	<18 y with suspected pneumonia	282	CXR
Chavez, 2015 [50]	Prospective observational study	Nepal and Peru	<10 y with and unwell with respiratory symptoms	378	Comparison with WHO pneumonia case algorithm
Ellington, 2017 [51]	Prospective observational study	Peru	2-59 mo with respiratory complaints	1300	CXR
Hajalioghli, 2016 [52]	Prospective observational study	Iran	<10 y with suspected pneumonia and referred by paediatrician for chest CT	98	Chest CT
Lovrenski, 2016 [53]	Prospective observational study	Serbia	Two months to 18 y with suspected pneumonia	95	Nil gold standard, auscultation as comparator
Osman, 2020 [54]	Prospective observational study	Egypt	<18 y with suspected pneumonia	90	Chest x-ray
Phung, 2020 [55]	Prospective observational study	Vietnam	Two months to 15 y with dyspnoea	81	Final diagnosis*
Saraya, 2017 [56]	Prospective observational study	Egypt	<10 y with suspected pneumonia	56	Chest CT
Yadav, 2017 [57]	Prospective observational study	India	2-59 mo with suspected pneumonia	118	CXR
Yan, 2020 [58]	Retrospective study	Inner Mongolia	<16 y with suspected pneumonia	959	Chest CT
Yilmaz, 2017 [59]	Prospective observational study	Turkey	One month to 18 y with suspected pneumonia	160	Nil gold standard, chest x-ray as comparator
**High-income country**					
Biagi, 2018 [12]	Prospective observational study	Italy	0-24 mo with bronchiolitis with suspicion of concomitant pneumonia	87	Final diagnosis*
Caiulo, 2011 [60]	Prospective observational study	Italy	1-16 mo with bronchiolitis	52	Clinical
Basile, 2015 [61]	Prospective observational study	Italy	<24 mo admitted with signs and symptoms of suspected bronchiolitis according to AAP	106	Clinical
Ambroggio, 2016 [62]	Prospective observational study	USA	Three months to 18 y with suspected pneumonia	132	Chest CT
Boursiani, 2017 [63]	Prospective observational study	Greece	Six months to 12 y with suspected pneumonia	66	Final diagnosis*
Caiulo, 2013 [64]	Prospective observational study	Italy	12 mo to 16 y with suspected pneumonia	102	Final diagnosis*
Claes, 2017 [65]	Prospective observational study	Belgium	<16 y with suspected pneumonia	143	CXR
Copetti, 2008 [66]	Prospective observational study	Italy	Six months to 16 y with suspected pneumonia	79	CXR or CT when clinically indicated
Esposito, 2014 [67]	Prospective observational study	Italy	One month to 14 y with suspected pneumonia	103	CXR
Ho, 2015 [68]	Retrospective study	Taiwan	<16 y with suspected pneumonia	163	CXR
Ianniello, 2016 [69]	Retrospective study (cohort or case-control)	Italy	3-16 y with cough and fever	84	CXR and clinical
Iorio, 2018 [70]	Retrospective study (cohort or case-control)	Italy	<10 y with a final diagnosis of pneumonia who had CXR and LUS within 24hrs of each other	47	CXR
Iuri, 2009 [71]	Prospective observational study	Italy	<17 y with suspected pneumonia	28	CXR
Lissaman, 2019 [72]	Prospective observational study	Australia	One month to 18 y with suspected pneumonia	97	CXR
Lai, 2015 [73]	Retrospective study (cohort or case-control)	Taiwan	Children with suspected pneumonia	236	Chest CT
Man, 2017 [74]	Retrospective study	Romania	<18 y with suspected pneumonia	81	CXR and clinical
Reali, 2014 [75]	Prospective observational study	Italy	Children with suspected pneumonia	81	Final diagnosis*
Samson, 2018 [76]	Prospective observational study	Spain	<15 y with suspected pneumonia	200	CXR
Shah, 2013 [77]	Prospective observational study	USA	<21 y with suspected pneumonia	200	CXR
Urbankowska, 2015 [78]	Prospective observational study	Poland	One month to 18 y with suspected pneumonia	106	CXR
Zhan, 2018 [79]	Prospective observational study	Denmark	<15 y with suspected pneumonia	82	CXR

CT – computerized tomography, CXR – chest x-ray, WHO – World Health Organization, AAP – American Academy of Pediatrics, mo – months, y – years

*Final diagnosis – a diagnosis of bacterial pneumonia made by an experienced paediatrician or pulmonologist, blinded to LUS findings, based on clinical presentation, laboratory tests, CXR, and clinical course.

The systematic reviews included a total of 32 studies, five of which were from LMICs (Peru [50,51], Egypt [56], India [57], and Turkey [59]). Most studies were conducted in emergency department or inpatient settings in HICs. Most studies used a reference standard for pneumonia which encompassed clinical and/or CXR features; however, computed tomography (CT) chest was the reference standard in a subset of patients in three studies [11,43,66].

The pooled sensitivity of LUS for pneumonia diagnosis was consistently high in all systematic reviews (range = 93%-96.7%), while pooled specificity was slightly lower (range = 87%-96.1%). Compared with CXR, LUS had higher sensitivity in identifying lung consolidation and had a high negative predictive value for pneumonia [11,43,44,48]. LUS was able to identify consolidation of less than 0.5-1 cm, which are often not distinguishable on CXR and may not represent true pneumonia. As a result, the specificity of LUS is reduced when comparing with CXR as a reference standard. One study which performed chest CT, CXR, and LUS, found high concordance between LUS and CT chest in identifying pneumonia (as determined by clinical course) when CXR was normal [66,71]. LUS was superior to CXR and comparable to CT scan in its ability to identify and characterise pleural effusions [71].

Factors which may impact diagnostic accuracy of LUS include operator experience, presence of comorbidities, and patient age. LUS studies of diagnostic accuracy have primarily been conducted in children without significant comorbidities. A study from the USA evaluated children with suspected pneumonia and comorbidities such as malignancy, who required a CT chest as part of their clinical care [62]. The accuracy of LUS using CT chest as a reference, was significantly lower than reported in other studies and similar to CXR (for consolidation, LUS sensitivity = 63% (95% CI = 0.34-0.85), specificity = 75% (95% CI = 0.58-0.87)). LUS had lower specificity compared with CXR for interstitial disease *(P <* .01), pleural effusion (*P* = 0.04), and consolidation (*P* = 0.26) in this patient population [62]. Three systematic reviews [11,42,44] performed sub-analysis of LUS diagnostic accuracy based on operator experience and found that accuracy improved with experience, but that sensitivity and specificity were still moderate to high in novice sonographers following variable periods of training (pooled sensitivity = 80%-95%, pooled specificity = 91%-96%). There was limited reporting of diagnostic accuracy based on age. One systematic review [44] identified a slightly better sensitivity and specificity in studies with a mean age of participants aged over five years, compared with studies with a mean age of less than five years. On the other hand, Pereda et al. [11] reported better diagnostic accuracy in neonates than in older children.

All systematic reviews reported heterogeneity between studies as a consistent limitation, including in operator experience, ultrasound technique, equipment utilised, and sonographic criteria for pneumonia.

#### Role of lung ultrasound in diagnosis

The characteristics, aims, and findings of the five studies evaluating LUS in diagnosis of severe pneumonia are presented in **Table 5**.

**Table 5:** Lung ultrasound studies

Setting	Study	Country	Study population, participants (n)	Study aim	Relevant findings
**Role of Lung Ultrasound in Diagnosis**					
LMIC	Pervaiz, 2018 [80]	Peru	<5 y, excluding neonates with CAP (n = 832)	Evaluated prediction models for clinical pneumonia with lobar consolidation +/− effusion, and included LUS in diagnostic algorithm	191 of the 832 children recruited met both clinical criteria of WHO defined pneumonia (2014) and radiological criteria. Consolidation on LUS added value to a clinical prediction model for radiographically confirmed clinical pneumonia, alone (AUC = 0.82, 95% CI = 0.78-0.85) or in addition to pulse oximetry and lung auscultation.
LMIC	Ozkaya, 2019 [81]	Turkey	<18 y, excluding neonates with respiratory distress (n = 145)	Prospective observational study evaluating concordance of ED diagnosis, final diagnosis, and Point-Of-Care (POC) LUS diagnosis performed by a single experienced sonographer, in a subset of children presenting with undifferentiated respiratory distress	LUS was able to diagnose cause of respiratory distress with high sensitivity and specificity for pneumonia (81.4% and 100%) using final clinical diagnosis as reference standard. Concordance between ED physician and LUS was high (kappa value 0.8). Similar high concordance was reported for diagnosis of acute bronchiolitis, asthma and croup, however, the method of LUS diagnosis of these conditions was not clear. Feasibility in this study was reported in time taken to perform LUS and make a diagnosis (mean time to LUS was 6.07 ± 3.92 min; time to sonographic diagnosis was shorter than to ED diagnosis: 29.32 ± 15.71 min (*P* < 0.001).
LMIC	Hegazy, 2020 [82]	Egypt	<18 y, excluding neonates with respiratory distress (n = 63)	Cross-sectional study evaluating bedside ultrasound performed by experienced operator in the emergency department to diagnose undifferentiated acute respiratory distress, using clinical diagnosis as reference standard.	31 children had clinical diagnosis of pneumonia. LUS had a higher sensitivity and specificity for pneumonia diagnosis (LUS = 93.5% sensitivity, 96.9% specificity) compared with CXR. LUS was able to identify pleural effusion and pneumothorax with 98 and 100% NPV respectively
LMIC	Chavez, 2015 [50]	Nepal and Peru	<10 y, excluding neonates with respiratory symptoms (further classified according to WHO defined pneumonia/severe pneumonia or no pneumonia) (n = 378)	Case-control study evaluating feasibility of portable LUS performed by GPs as well as agreement between WHO defined pneumonia and consolidation on LUS. GPs underwent 7 d of training (including 3 d theoretical and 4 d ward-based)	39% of children had a discrepancy in diagnosis between lung US consolidation and WHO classified pneumonia. Of children classified as WHO pneumonia (n = 169), 35% had normal US and 23% had lung consolidation. Of children with WHO severe pneumonia (n = 12/169), 33% had lung consolidation on LUS. Misclassification remained high when LUS findings of any abnormality were included in definition of pneumonia (28%). Children with respiratory symptoms without WHO pneumonia were just as likely to have lung consolidation on US.
HIC	Berce, 2019 [83]	Slovenia	<16 y, excluding neonates with CAP, excluded ICU patients and any patient with condition predisposing to pneumonia, (n = 147)	Evaluated association between LUS findings and aetiological diagnosis (viral, bacterial or atypical bacterial) Aetiology was assigned based on a combination of CXR alveolar changes, blood infection markers, blood culture results and nasopharyngeal aspirate and	Association between presumed bacterial origin, and large and solitary consolidations, as well as viral infection and multiple consolidations: Median diameter of consolidation in presumed viral pneumonia: 15 mm, compared to 20 mm in atypical bacterial CAP (*P* = 0.05) and 30 mm in bacterial CAP (*P* < 0.001). Multiple consolidations more common in viral pneumonia (65.4% viral pneumonia vs 17.3% bacterial pneumonia (*P* < 0.001)). This study provided detailed definitions to classify viral vs bacterial infections but is limited by the challenges of accurately identifying aetiology.
**Association between LUS abnormalities and outcomes**					
LMIC	Özkaya, 2020 [84]	Turkey	Infants presenting to ED with bronchiolitis (n = 76)	Prospective study evaluating LUS predictive value for hospital admission	LUS was consistent with bronchiolitis in 74/76 infants presenting to ED with bronchiolitis and 41/44 admitted patients. Unclear whether LUS conferred additional benefit to bronchiolitis severity score in predicting admission to hospital.
HIC	Bueno- Campana, 2019 [85]	Spain	<6 mo with suspected bronchiolitis (n = 145)	Role of LUS in predicting need for respiratory support in bronchiolitis. LUS performed by experienced operator	59/145 infants required respiratory support. A score based on clinical variables *and* LUS findings on the 3rd day of respiratory distress showed a moderate predictive ability, with good NPV. Identification of at least one posterior consolidation >1 cm was the main factor associated with non-invasive ventilation (RR = 4.4; 95%CI = 1.8-10.8) Note: other studies evaluating children with bronchiolitis have not found consolidation to be a common feature
HIC	Supino, 2019 [86]	Italy	<24 mo with bronchiolitis (n = 76)		32/76 infants required CPAP or HFNC. Higher LUS score was associated with the need for respiratory support (*P* = 0.003), as was bronchiolitis clinical severity score. Unclear benefit of LUS over clinical assessment alone.
HIC	Di Mauro, 2020 [87]	Italy	<24 mo with bronchiolitis (n = 83)		27/83 required oxygen during admission. LUS score was found to be associated with the need for supplemental oxygen during hospitalisation (OR = 2.2; 95% CI = 1.5-3.3; *P* < 0.0001)
HIC	Guerra, 2016 [88]	Italy	Three months to 16 y febrile children with respiratory distress (n = 222)	LUS V CXR in children with CAP including severe pneumonia, with secondary evaluation of LUS value in predicting fever duration	Presence of pleural effusion or “liver-like” consolidation pattern on LUS was associated with lack of fever defervescence at 48 h
HIC	Musolino, 2019 [89]	Italy	<18 y with clinical diagnosis of CAP and underwent LUS (n = 101)	Prospective study evaluating LUS abnormality at admission at 48 h in children who develop complicated pneumonia vs those who do not	13% of participants had complicated pneumonia, defined as requirement for PICU, CPAP, invasive ventilation, pleural drainage, or hospitalisation for >10 d. More severe LUS findings at baseline, such as large (>5cm) subpleural pulmonary parenchymal lesions and pleural effusion, were associated with development of complicated CAP.
HIC	Chen, 2017 [90]	Taiwan	6 mo to 18 y admitted with diagnosis of CAP and underwent lung US within 48 h (n = 142)	Retrospective evaluation of association between Lung US findings within 48 h of admission and clinical outcome	9% of participants required tube thoracotomy and 20% required ICU admission. LUS findings of fluid bronchogram, multifocal involvement (≥2-3 lobes) and pleural effusion on early ultrasound, were associated with adverse outcomes including longer length of stay. There were wide CI for these outcomes in multiple logistic regression.
HIC	Buonsenso, 2020 [91]	Italy	<18 y, excluding neonates with CAP (n = 121)	Prospective evaluation of LUS findings in children requiring surgical intervention for pneumonia, comparing with children conservatively managed	18% of children required surgical intervention. Paediatrician performed LUS at 12 h into admission correlated with final surgical decision in 21 out of 22 cases where surgical intervention was required, identifying large pleural effusion, necrotizing pneumonia and indirect signs of bronchopleural fistula. LUS was performed routinely in this hospital and may have influenced decision making. LUS performed better than both CXR and CT scan in identifying large and complicated effusions.
**Practical considerations of LUS (feasibility, inter-operator reliability)**					
LMIC	Pervaiz, 2019 [92]	Peru	3-35-mo-olds with WHO defined CAP (part of PCV10 impact study) (n = 9051)	Evaluated training of General Practitioners in LUS as part of broader vaccine impact study. five-month training program with significant financial support involved seven days practical and didactic training (12 zone technique), expert review of LUS images, local expert supervision followed by refresher training to achieve >85% accuracy in diagnosis of primary endpoint pneumonia.	Sustained levels of high agreement for diagnosis of PEP (κ>0.78), agreement was higher in children with danger signs. The training was resource intensive and expensive so may limit application in resource limited settings. This study provides criteria for PEP on LUS which requires further diagnostic validation to be widely implemented.
LMIC	Nadimpalli, 2019 [93]	South Sudan	<14 y with clinical signs of LRTI (n = 168)	Feasibility study. Clinical officers (novice, non-clinician) received 12-h field-based training didactic and practical components, six zone technique. Images were reviewed by expert sonologists.	High concordance between novice and expert sonologists for diagnosis of bacterial pneumonia (κ = 0.73, 95% CI = 0.63-0.82) in this short term follow up period and high specificity for diagnosis of bacterial pneumonia/lung consolidation on LUS (98%, 95% CI = 95-99), and sensitivity 69% (95% CI = 58%-78%) compared to expert reviewer LUS diagnosis
LMIC	deSouza, 2019 [94]	Brazil	<14 y with clinically suspected bacterial pneumonia (n = 23)	Assessed LUS inter-operator reliability between a resident who received 14 h of practical training in point of care LUS (six zone) and an expert sonologist, they also compared LUS to CXR	This training resulted in substantial agreement for consolidation (κ = 0.635, 95% CI = 0.532-0.738). In this small sample, there was high sensitivity of LUS compared to CXR for lung consolidation, but low specificity of LUS compared to CXR (secondary outcome).
LMIC	Correa, 2018 [95]	Peru	<5 y with lobar pneumonia on CXR (n = 21)	Small proof of concept study on a computerised algorithm that allows automatic recognition of pulmonary infiltrates from LUS images obtained by non-expert technicians who received training in obtaining linear LUS images (12 zone, details of training not provided).	Achieved a sensitivity of 90.09% and specificity of 100%. when compared to visual recognition by an expert analyst
LMIC	Chavez, 2015 [50]	Nepal and Peru	<10 y with respiratory symptoms (n = 378)	Two GPs trained in LUS with a standardised training course over seven days, including three days theoretical delivered by an experienced radiologist, and four days of hands-on training in a paediatric ward	Average time including setup and video recording was 6.4 +/− 2.2 min (did not perform oblique or transverse views). Interobserver agreement was high: κ = 0.79, 95% CI = 0.73-0.81. Supine position only (no oblique or transverse views), 6 sections evaluated with portable, laptop sized device. No issues with implementation reported.
HIC	Jones, 2016 [96]	USA	<21 y of age presenting to paediatric ED with clinical suspicion of having pneumonia requiring CXR for evaluation (n = 378)	Randomised controlled trial comparing point of care LUS performed by paediatric emergency physicians with varying degrees of experience, with CXR	POC LUS resulted in 38% reduction in CXR use, no statistically significant differences with respect to missed pneumonia, unscheduled health care visits, rates of consolidated pneumonia visible radiographically, rates of antibiotic use at the index ED visit (37.9%, investigational arm; 27.3%, control arm), overall median ED LOS, and hospital admission rates between groups. POC LUS resulted in shorter ED length of stay and cost savings.
HIC	Harel-Sterling, 2019 [97]	Canada	<18 y with suspected pneumonia (n = 202)	Retrospective matched cohort study comparing POS LUS performed by credentialed paediatric emergency physician, with CXR.	Significant reduction in ED length of stay in LUS group and significant cost savings (facility fees and physician billing), CXR group were more likely to have blood test than US group, no significant difference between groups in discharge diagnosis of pneumonia, number of patients given antibiotics or return ED visits within 3 weeks.
HIC	Ambroggio, 2016 [62]	USA	3 mo – 18 y with suspected pneumonia, and had a CT scan for a clinical reason (n = 132 (36 had CT scan))	Prospective cohort study evaluating interrater reliability of CXR and LUS and diagnostic accuracy of CXR and LUS compared with chest CT scan.	Agreement for consolidation on LUS was moderate (0.55; 95% CI = 0.40-0.70), and on CXR was poor (0.36; 95% CI = 0.21-0.51). For interstitial disease, agreement was poor on LUS (0.32; 95% CI = 0.17-0.47), and substantial for CXR (0.63; 95% CI = 0.47-0.77). LUS performed by multiple sonographers and interpreted by multiple paediatric radiologists to improve applicability to clinical settings.

LUS – lung ultrasound, POC LUS – point-of-care lung ultrasound, NPV – negative predictive value, HFNC – high flow nasal cannulae, ED – emergency department, PICU – paediatric intensive care unit, CPAP – continuous positive airway pressure, PEP – primary endpoint pneumonia (presence of pleural effusion), LMIC – low- and middle-income country, HIC – high-income country, LUS – lung ultrasound, CXR – chest x-ray, κ – kappa value, RR – risk ratio, OR – odds ratio, CI – confidence interval, GP – general practitioner, CAP – community-acquired pneumonia, ED – emergency department, mm – millimetres, AUC – area under curve, mo – months, y – years

Four out of five studies evaluating LUS in diagnosis of severe pneumonia were conducted in LMICs [50,80-82]. Two of these, one in Egypt and one in Turkey, evaluated the diagnostic utility of LUS in children presenting with undifferentiated respiratory symptoms, and found that LUS diagnosis of pneumonia, pleural effusion, and pneumothorax correlated well with a final physician diagnosis. A total of 208 children were enrolled in the studies and an experienced operator performed the bedside LUS [50,51]. Another study from Turkey also reported ED physician diagnosis to have similar accuracy to LUS [84]. A larger study of 832 children with respiratory symptoms in Peru found that LUS predicted pneumonia with greater accuracy than physicians did based on clinical signs and symptoms, CXR, pulse oximetry, and chest auscultation [80].

LUS pneumonia correlated poorly with WHO-defined pneumonia [50,80,83]. In a study of 378 children with respiratory symptoms conducted in Nepal and Peru [50], the proportion of children with consolidation on LUS was similar in children with WHO-defined pneumonia and children with respiratory symptoms not meeting WHO pneumonia criteria (23 vs 21%, *P* = 0.68). Consolidation occurred in 33% of children with WHO-defined severe pneumonia (n = 12). A study conducted in Slovenia [83] with 378 children correlated large and solitary consolidation on LUS with presumed bacterial pneumonia, and a pattern of small, multiple consolidations with viral pneumonia. The probable aetiology was assigned based on a combination of CXR, blood infection markers, blood cultures, and nasopharyngeal aspirate PCR.

#### Lung ultrasound and outcomes

No study directly assessed the impact of the use of LUS on patient outcomes. Eight studies evaluated the association between LUS abnormalities and outcomes, one of which [84] was from an LMIC (Turkey). One study [88] found an association between sonographic lung consolidation and lack of fever defervescence at 48 hours. Four observational studies (three from countries in Europe and one from Turkey) comprising 380 infants with bronchiolitis [84-87] found associations between LUS abnormalities and the need for respiratory support or hospital admission, but there was no clear benefit described over clinical assessment alone.

In 364 children with suspected community-acquired pneumonia in Italy and Taiwan, LUS was used to identify surgical complications [89-91]. LUS was performed early in admission and identified signs of evolving complications of pneumonia before they were clinically apparent. Pleural effusion on initial ultrasound was associated with risk of complicated pneumonia, adverse outcome, or surgical intervention. Other LUS abnormalities were less consistently associated with outcomes. LUS performed better than both CXR and CT scan in identifying large and complicated effusions.

#### Feasibility and practical considerations for low- and middle-income country settings

Nine studies [50,62,92-97] assessed feasibility including inter-operator reliability of LUS as a primary or secondary outcome, six of which were conducted in LMICs. Four studies, including a large vaccine impact study with 9051 participants, assessed the validity of training novice clinicians in point-of-care ultrasound for suspected pneumonia in LMICs. Training varied in intensity (12 hours to seven days plus ongoing supervision and refresher training). All training resulted in good inter-rater reliability between expert and novice LUS operators for consolidation (range of kappa values = 0.635 to 0.78). The longer and more intensive training program resulted in sustained levels of high agreement for LUS findings over two-year follow-up; however, the program was resource intensive and costly [92]. Two small studies conducted in HICs [96,97] compared feasibility of point-of-care LUS performed by experienced paediatric emergency physicians with CXR. The studies reported no missed pneumonia or increase in adverse events, shorter lengths of stay in the emergency department. and associated health care cost savings. A study conducted in the USA [62] assessed the reliability of LUS when performed by multiple operators and interpreted by multiple paediatric radiologists; inter-operator agreement for consolidation was lower than other studies (ĸ = 0.55; 95% CI = 0.40-0.70) .

## DISCUSSION

Although CXR is widely used for child pneumonia management, there is a lack of evidence to inform the optimal or pragmatic use of CXR for children presenting with possible pneumonia. Most existing research explored CXR as an independent tool in pneumonia diagnosis or evaluated association between CXR findings and patient outcomes, with significant limitations identified. Research evaluating LUS has focused mainly on diagnostic accuracy, and utility and feasibility are yet to be determined. Given the challenge of lack of a ‘gold standard’ for pneumonia diagnosis, ascertaining the precise diagnostic accuracy of CXR and LUS for pneumonia remains elusive.

While CXR had suboptimal performance as an independent tool in severe pneumonia, there was little high-quality evidence to support, refute, or guide the use of CXR as it is conventionally interpreted – with consideration to clinical and contextual variables such as age, comorbidities, duration of illness, and prior antibiotic treatment. Besides for diagnostic clarification, CXR is often used to investigate treatment failure and identify complications, comorbidities, or alternate diagnoses. However, there were no studies evaluating these indications for CXR. The lack of clear evidence is reflected in the substantial variation in national and international guidelines for use of radiographs in severe pneumonia in HIC and LMIC settings [6].

Despite many evidence gaps, there were a few consistent themes in the literature. Radiographic “dense” consolidation was associated with mortality in several studies from resource-limited settings. This has not been a convincing finding globally, and a recent systematic review including a large proportion of studies from HICs identified multi-lobar consolidation to be more predictive of adverse outcomes [23]. The variability may reflect differences in local pneumonia-related epidemiology, vaccination status, patient co-morbidities, and availability of timely and appropriate treatment, and underscores the need for individualised interpretation and management of CXR abnormalities. Moreover, while positive CXR findings may be helpful in confirming a diagnosis, studies consistently identified a normal CXR in a small but significant proportion of children who were likely to have “true” severe pneumonia. Additionally, CXR was unable to reliably determine whether pneumonia is of bacterial or viral origin. There is wide inter-observer variability in the interpretation of CXR abnormalities, particularly with less experienced clinicians and more obscure findings, and the use of a standardised format for interpretation improved concordance.

LUS is superior to CXR in the detection of lung consolidation and pleural effusion, with high sensitivity consistently identified in several meta-analyses. Specificity was also high, but contingent upon the reference standard utilised for pneumonia, possibly making it lower in children with comorbidities. There is currently no consensus on the sonographic criteria for pneumonia or on an optimal scanning protocol. Small studies indicate LUS may have diagnostic capabilities in children with undifferentiated respiratory distress. Inter-operator reliability of LUS was influenced by operator experience, yet preliminary research on its feasibility in LMICs indicates that good diagnostic performance can be achieved following training of novice operators. Despite widespread availability of point of care ultrasound devices in HIC settings, LUS is not widely utilised as a first line investigation for pneumonia [98], particularly in settings where clinicians have no prior experience with its use. Barriers cited by clinicians include a lack of confidence in skills and challenge of maintaining expertise over time [67]. While LUS is less costly to perform than CXR [65], the financial and personnel costs of training and ongoing support to maintain expertise in the longer term may be significant.

### Strengths and limitations

Our scoping review was deliberately comprehensive in order to understand the range of relevant research. Therefore, we were able to obtain a clear picture of the strengths and limitations of the available research and identify significant gaps in knowledge. For both LUS and CXR studies, the lack of an optimal reference standard for pneumonia is a major limitation for evaluation. Moreover, both modalities lack a standardised radiological definition of pneumonia for clinical settings, hindering our ability to draw robust conclusions. By excluding CXR studies from HICs, we acknowledge there may have been data applicable to resource-limited settings which we could not include in our study. Similarly, we only evaluated recent literature from the last two decades, which may have excluded older studies relevant to informing and establishing the current practice of CXR. Any conclusions drawn regarding the practical utility of CXR and LUS is based on limited data as high-quality evidence was not available and for some outcomes there were no relevant studies.

### Practical application

**T**he limited diagnostic accuracy, combined with challenges of obtaining high quality images and need for expert interpretation do not support large scale programmatic use of CXR for pneumonia or specific recommendations for use in health facilities in resource-limited settings. CXR is unlikely to alter the initial case management of children with severe pneumonia. There is a paucity of research into the utility of CXR in specific contexts, such as exploring alternative diagnoses and investigating sudden deterioration or poor response to treatment. In such cases, CXR may identify complications such as pleural effusion, lung abscess, pneumothorax, pneumatoceles, or characteristic features of a specific aetiology such as tuberculosis. There is currently insufficient evidence to provide recommendations for the use of LUS in the case management of children with pneumonia. The use of LUS in the management of complicated pneumonia has already been established and may have a role in improving accuracy of a clinical diagnosis of pneumonia; further exploration of its utility and feasibility in low resource settings is needed.

## CONCLUSIONS

Pneumonia is a heterogenous entity, encompassing a range of aetiologies with a clinical course dependant on host factors, clinical context, and access to timely and appropriate treatment. There is significant discrepancy between the evidence for utility of CXR and its widespread and ongoing use for pneumonia. High-quality, pragmatic research is needed to understand its role and perceived benefits, including the identification of alternate diagnoses or comorbidities in children presenting with severe pneumonia, investigation of treatment failure, or identification of complications.

LUS has the potential to improve diagnostic capabilities in low resource settings, with the benefit of greater sensitivity for pneumonia than CXR. However, there remain significant gaps in knowledge. High-quality, prospective studies are warranted in resource limited settings to explore LUS’s role in improving the accuracy of clinical diagnosis as well as predicting and managing complications of pneumonia, and on the benefits and risks of this imaging tool. Although LUS findings in pneumonia are well described, consensus is required regarding scanning protocols and diagnostic criteria. Future feasibility studies should evaluate barriers to implementation and uptake of LUS by clinicians, with longitudinal follow up to understand the maintenance needs for both equipment and technical skills over time.

Children with co-morbidities which increase vulnerability to pneumonia-related mortality (such as malnutrition, HIV, and congenital heart disease) should be included in research of imaging tools to promote early and accurate diagnosis in this high-risk population.

## Additional material


Online Supplementary Document.
