# Supplementary material for: The utility of chest x-ray and lung ultrasound in the management of infants and children presenting with severe pneumonia in low-and middle-income countries: A pragmatic scoping review
Source: J Glob Health. 2022 Dec 23;12:10013. doi: 10.7189/jogh.12.10013 (PMC9789364; doi:10.7189/jogh.12.10013)
Supplement: Online Supplementary Document. [file jogh-12-10013-s001.pdf]

Supplemental Material

Appendix S1: Example Search Strategy (used for MEDLINE)

1 exp *Pneumonia/
2 ((respiratory adj3 infection*) or pneumonia or pneumonias or lung-inflammation* or lobitis or nonspecific-inflammatory-lung-disease* or peripneumonia or pleuropneumonia or pleuropneumonitis or pneumonic-lung* or pneumonic-pleurisy or pneumonic-pleuritis or pneumonitides or pneumonitis or pulmonal-inflammation* or pulmonary-inflammation* or pulmonic-inflammation* or bronchiolitis).tw,kf.
3 *Pneumococcal Infections/
4 1 or 2 or 3
5 (x-ray* or xray* or imaging or radiolog* or cxr or radiograp*).tw,kf,hw.
6 *radiology/ or *radiography/ or exp *radiography, thoracic
7 5 or 6
8 dg.fs.
9 exp *Ultrasonography/
10 (lung-us or lus).tw,kf.
11 8 or 9 or 10
12 *diagnosis/ or *clinical decision-making/ or *delayed diagnosis/ or *diagnosis, differential/ or *early diagnosis/
13 *"Severity of Illness Index"/
14 exp *Hospitalization/
15 *child, hospitalized/ or *inpatients/
16 *Tertiary Care Centers/
17 *"referral and consultation"/ or *secondary care/ or *tertiary healthcare/
18 exp *Mortality/ or *Morbidity/
19 *death/ or exp *infant death/
20 (co or mo).fs.
21 *Treatment Outcome/
22 *intensive care units/ or *intensive care units, pediatric/ or *respiratory care units/ or *critical care/
23 (Diagnosis or decision-making or severe or severity or inpatient* or admission* or mortalit* or death* or died or surviv* or fatal* or hospitali#ation or hospitali#ed or refer* or morbidit* or length-of-stay or icu or picu or outcome* or ventilation or ventilator* or critical-care).tw,kf.
24 12 or 13 or 14 or 15 or 16 or 17 or 18 or 19 or 20 or 21 or 22 or 23
25 (infan* or toddler* or pre-schooler* or preschooler* or kinder or kinders or kindergarten* or kinder-aged or boy or boys or girl or girls or child or children or childhood or pediatric* or paediatric* or school-age* or schoolage* or schoolchild* or schoolgirl* or schoolboy*).af.
26 developing countries/
27 (austere or (limited adj2 resource*) or (low adj2 resource*) or (transitioning adj econom*) or (third adj world) or LMIC or LMICs or (lami adj countr*) or (transitional adj countr*) or (low adj gdp) or (low adj gnp) or (low adj gross adj domestic) or (low adj gross adj national) or ((emerging or developing or (low adj income) or (middle adj income) or (low adj3 middle) or underdeveloped or under-developed or (less* adj developed) or underserved or under-served or deprived or poor*) and (countr* or nation*1 or econom* or population or world))).tw,kf.
28 exp africa/
29 americas/ or exp caribbean region/ or exp central america/ or latin america/ or mexico/ or exp south america/
30 europe/ or exp europe, eastern/ or exp transcaucasia/
31 antarctic regions/ or exp atlantic islands/ or exp indian ocean islands/ or exp pacific islands/
32 New Guinea/
33 asia/ or exp asia, central/ or asia, southeastern/ or borneo/ or cambodia/ or east timor/ or indonesia/ or laos/ or malaysia/ or mekong valley/ or myanmar/ or philippines/ or thailand/ or vietnam/ or asia, western/ or bangladesh/ or bhutan/ or india/ or middle east/ or afghanistan/ or iran/ or iraq/ or jordan/ or lebanon/ or

oman/ or saudi arabia/ or syria/ or turkey/ or yemen/ or nepal/ or pakistan/ or sri lanka/ or far east/ or china/ or tibet/ or exp korea/ or mongolia/

34 (Afghanistan or Albania or Algeria or Angola or Antigua or Argentina or Armenia* or Aruba or Azerbaijan or Bahrain or Bangladesh or Barbados or Barbuda or Belarus or Byelarus* or Byelorussian or Belorussian or Belorus* or Belize or Benin or Bhutan or Bolivia or Bosnia or Botswana or Brasil or Brazil or Bulgaria or (Burkina adj Fas*) or (Upper adj Volta) or Burma or Burundi or Cambodia or Khmer or Kampuchea or Cameron* or Cameroon* or (Cape adj Verde) or (Cabo adj Verde) or (Central adj African adj Republic) or Chad or Chile or China or Colombia or Comoros or (Comoro adj Island*) or Comores or Mayotte or Congo or Kongo or (Cook adj Island*) or (Costa adj Rica) or (Cote adj D'ivoire) or Croatia or Cuba or Cyprus or Czech* or Djibouti or Dominica or Dominican or (East adj Timor) or (East adj Timur) or Ecuador or Egypt or El-Salvador or (Equatorial adj Guinea) or Eritrea or Estonia or Ethiopia or Fiji or (French adj Somaliland) or Futuna or Gabon or (Gabonese adj Republic) or Gambia or Gaza or (Georgia* adj Republic) or Ghana or Grenada or Guam or Guatemala or Guinea or Guiana or Guyana or Haiti or Herzeg* or Hercegovina or Honduras or Hungary or India or Indonesia or Iran or Iraq or (Ivory adj Coast) or Jamaica or Jordan or Kazakh* or Kenya or Kiribati or Korea or Kosovo or (Kyrgyz adj Republic) or Kyrgyzstan or Kirghizia or Kirghiz or Kirgizstan or Laos or (Lao* adj2 Democratic adj Republic) or (Lao* adj PDR) or Latvia or Lebanon or Lesotho or Basutoland or Liberia or Libya or Lithuania or Macedonia or Madagascar or (Magalasy adj Republic) or Malawi or Malay* or Sabah or Sarawak or Maldives or Mali or (Marshall adj Island*) or Mauritania or Mauritius or (Agalega adj Island*) or Mexico or Micronesia or Moldov* or Mongolia or Montserrat or Montenegro or Morocco or Ifni or Mozambique or Myanma* or Namibia or Nauru or Nepal or (Netherlands adj Antilles) or (Dutch adj Antilles) or (New adj Guinea) or (New adj Caledonia) or Nicaragua or Niue or Niger or Nigeria or (Northern adj Mariana adj Island*) or Nyasaland or Oman or Pakistan or Palau or Panama or (Papua adj New adj Guinea) or PNG or Palestine or Paraguay or Peru or Philipines or Philippines or Phillipines or Phillippines or Poland or (Puerto adj Rico) or Yemen or Romania or Roumania or Rumania or Russia* or Rwanda or Ruanda or (Saint adj Kitts) or (St adj Kitts) or Nevis or (Saint adj Vincent) or (St adj Vincent) or Grenadines or Samoa* or (Navigator adj Island*) or (Saint adj Lucia) or (St adj Lucia) or (Saint adj Helena) or (St adj Helena) or (Sao adj Tome) or (Saudi adj Arabia) or Senegal or Serbia or Seychelles or (Sierra adj Leone) or Slovenia or Slovak* or (South adj Africa) or (Solomon adj Island*) or Somalia or (Sri adj Lanka) or Ceylon or Sudan or Surinam* or Swaziland or Syria or Tajikistan or Tadzhikistan or Tadjikistan or Tadzhik or Tanzania or Thailand or Tibet or Timor-Leste or Togo or (Togolese adj Republic) or Tokelau or Tonga or Trinidad or Tobago or Tunisia or Turkey or Turkmenistan or Turkmen or Tuvalu or Uganda or Ukraine or Uruguay or Urundi or USSR or (Soviet adj Union) or "Union of Soviet Socialist Republics" or Uzbekistan or Vanuatu or (New adj Hebrides) or Venezuela or Vietnam or (Viet adj Nam) or (Wallis adj2 Futuna) or (United adj Arab adj Republic) or (West adj Bank) or (West adj Indies) or Yemen or Yugoslavia or Zaire or Zambia or Zimbabwe or Rhodesia).tw,kf.

35 (africa or americas or caribbean or (central adj America) or (latin adj America) or (south adj America) or (eastern adj Europe) or Transcaucasia or antarctic or (atlantic adj island*) or (indian adj ocean adj island*) or (pacific adj island*) or polynesia or (central adj asia) or (southeast* adj asia) or (south-east* adj asia) or borneo or mekong or (western adj asia) or (middle adj east) or (far adj east)).tw,kf.

36 26 or 27 or 28 or 29 or 30 or 31 or 32 or 33 or 34 or 35

37 4 and 7 and 24 and 25 and 36

38 4 and 11 and 24 and 25

39 37 or 38

40 limit 39 to (english language and yr="2000 -Current"

41 exp *Pneumonia/

42 ((respiratory adj3 infection*) or pneumonia or pneumonias or lung-inflammation* or lobitis or nonspecific-inflammatory-lung-disease* or peripneumonia or pleuropneumonia or pleuropneumonitis or pneumonic-lung* or pneumonic-pleurisy or pneumonic-pleuritis or pneumonitides or pneumonitis or pulmonal-inflammation* or pulmonary-inflammation* or pulmonic-inflammation* or bronchiolitis).tw,kf.

43 *Pneumococcal Infections/

44 41 or 42 or 43

45 (x-ray* or xray* or imaging or radiolog* or cxr).tw,kf,hw.

46 *radiology/ or *radiography/ or exp *radiography, thoracic/

47 45 or 46

48 dg.fs.

49 exp *Ultrasonography/

50 (lung-us or lus).tw,kf.

51 48 or 49 or 50
52 *diagnosis/ or *clinical decision-making/ or *delayed diagnosis/ or *diagnosis, differential/ or *early diagnosis/
53 *"Severity of Illness Index"/
54 exp *Hospitalization/
55 *child, hospitalized/ or *inpatients/
56 *Tertiary Care Centers/
57 *"referral and consultation"/ or *secondary care/ or *tertiary healthcare/ (26708)
58 exp *Mortality/ or *Morbidity/
59 *death/ or exp *infant death/
60 (co or mo).fs.
61 *Treatment Outcome/
62 *intensive care units/ or *intensive care units, pediatric/ or *respiratory care units/ or *critical care/
63 (Diagnosis or decision-making or severe or severity or inpatient* or admission* or mortalit* or death* or died or surviv* or fatal* or hospitali#ation or hospitali#ed or refer* or morbidit* or length-of-stay or icu or picu or outcome* or ventilation or ventilator* or critical-care).tw,kf.
64 52 or 53 or 54 or 55 or 56 or 57 or 58 or 59 or 60 or 61 or 62 or 63
65 (infan* or toddler* or pre-schooler* or preschooler* or kinder or kinders or kindergarten* or kinder-aged or boy or boys or girl or girls or child or children or childhood or pediatric* or paediatric* or school-age* or schoolage* or schoolchild* or schoolgirl* or schoolboy*).af.
66 developing countries/
67 (austere or (limited adj2 resource*) or (low adj2 resource*) or (transitioning adj econom*) or (third adj world) or LMIC or LMICs or (lami adj countr*) or (transitional adj countr*) or (low adj gdp) or (low adj gnp) or (low adj gross adj domestic) or (low adj gross adj national) or ((emerging or developing or (low adj income) or (middle adj income) or (low adj3 middle) or underdeveloped or under-developed or (less* adj developed) or underserved or under-served or deprived or poor*) and (countr* or nation*1 or econom* or population or world)))
68 exp africa/
69 americas/ or exp caribbean region/ or exp central america/ or latin america/ or mexico/ or exp south america/
70 europe/ or exp europe, eastern/ or exp Transcaucasia/
71 antarctic regions/ or exp atlantic islands/ or exp indian ocean islands/ or exp pacific islands/
72 New Guinea/
73 asia/ or exp asia, central/ or asia, southeastern/ or borneo/ or cambodia/ or east timor/ or indonesia/ or laos/ or malaysia/ or mekong valley/ or myanmar/ or philippines/ or thailand/ or vietnam/ or asia, western/ or bangladesh/ or bhutan/ or india/ or middle east/ or afghanistan/ or iran/ or iraq/ or jordan/ or lebanon/ or oman/ or saudi arabia/ or syria/ or turkey/ or yemen/ or nepal/ or pakistan/ or sri lanka/ or far east/ or china/ or tibet/ or exp korea/ or Mongolia/
74 (Afghanistan or Albania or Algeria or Angola or Antigua or Argentina or Armenia* or Aruba or Azerbaijan or Bahrain or Bangladesh or Barbados or Barbuda or Belarus or Byelarus* or Byelorussian or Belorussian or Belorus* or Belize or Benin or Bhutan or Bolivia or Bosnia or Botswana or Brasil or Brazil or Bulgaria or (Burkina adj Fas*) or (Upper adj Volta) or Burma or Burundi or Cambodia or Khmer or Kampuchea or Cameron* or Cameroon* or (Cape adj Verde) or (Cabo adj Verde) or (Central adj African adj Republic) or Chad or Chile or China or Colombia or Comoros or (Comoro adj Island*) or Comores or Mayotte or Congo or Kongo or (Cook adj Island*) or (Costa adj Rica) or (Cote adj D'ivoire) or Croatia or Cuba or Cyprus or Czech* or Djibouti or Dominica or Dominican or (East adj Timor) or (East adj Timur) or Ecuador or Egypt or El-Salvador or (Equatorial adj Guinea) or Eritrea or Estonia or Ethiopia or Fiji or (French adj Somaliland) or Futuna or Gabon or (Gabonese adj Republic) or Gambia or Gaza or (Georgia* adj Republic) or Ghana or Grenada or Guam or Guatemala or Guinea or Guiana or Guyana or Haiti or Herzeg* or Hercegovina or Honduras or Hungary or India or Indonesia or Iran or Iraq or (Ivory adj Coast) or Jamaica or Jordan or Kazakh* or Kenya or Kiribati or Korea or Kosovo or (Kyrgyz adj Republic) or Kyrgyzstan or Kirghizia or Kirghiz or Kirgizstan or Laos or (Lao* adj2 Democratic adj Republic) or (Lao* adj PDR) or Latvia or Lebanon or Lesotho or Basutoland or Liberia or Libya or Lithuania or Macedonia or Madagascar or (Magalasy adj Republic) or Malawi or Malay* or Sabah or Sarawak or Maldives or Mali or (Marshall adj Island*) or Mauritania or Mauritius or (Agalega adj Island*) or Mexico or Micronesia or Moldov* or Mongolia or Montserrat or Montenegro or Morocco or Ifni or Mozambique or Myanma* or Namibia or Nauru or Nepal or (Netherlands adj Antilles) or (Dutch adj Antilles) or (New adj Guinea) or (New adj Caledonia)

or Nicaragua or Niue or Niger or Nigeria or (Northern adj Mariana adj Island*) or Nyasaland or Oman or Pakistan or Palau or Panama or (Papua adj New adj Guinea) or PNG or Palestine or Paraguay or Peru or Philipines or Philippines or Phillipines or Phillippines or Poland or (Puerto adj Rico) or Yemen or Romania or Roumania or Rumania or Russia* or Rwanda or Ruanda or (Saint adj Kitts) or (St adj Kitts) or Nevis or (Saint adj Vincent) or (St adj Vincent) or Grenadines or Samoa* or (Navigator adj Island*) or (Saint adj Lucia) or (St adj Lucia) or (Saint adj Helena) or (St adj Helena) or (Sao adj Tome) or (Saudi adj Arabia) or Senegal or Serbia or Seychelles or (Sierra adj Leone) or Slovenia or Slovak* or (South adj Africa) or (Solomon adj Island*) or Somalia or (Sri adj Lanka) or Ceylon or Sudan or Surinam* or Swaziland or Syria or Tajikistan or Tadzhikistan or Tadjikistan or Tadzhik or Tanzania or Thailand or Tibet or Timor-Leste or Togo or (Togolese adj Republic) or Tokelau or Tonga or Trinidad or Tobago or Tunisia or Turkey or Turkmenistan or Turkmen or Tuvalu or Uganda or Ukraine or Uruguay or Urundi or USSR or (Soviet adj Union) or "Union of Soviet Socialist Republics" or Uzbekistan or Vanuatu or (New adj Hebrides) or Venezuela or Vietnam or (Viet adj Nam) or (Wallis adj2 Futuna) or (United adj Arab adj Republic) or (West adj Bank) or (West adj Indies) or Yemen or Yugoslavia or Zaire or Zambia or Zimbabwe or Rhodesia).tw,kf.
75 (africa or americas or caribbean or (central adj America) or (latin adj America) or (south adj America) or (eastern adj Europe) or Transcaucasia or antarctic or (atlantic adj island*) or (indian adj ocean adj island*) or (pacific adj island*) or polynesia or (central adj asia) or (southeast* adj asia) or (south-east* adj asia) or borneo or mekong or (western adj asia) or (middle adj east) or (far adj east)).tw,kf.
76 66 or 67 or 68 or 69 or 70 or 71 or 72 or 73 or 74 or 75
77 44 and 47 and 64 and 65 and 76
78 44 and 51 and 64 and 65
79 77 or 78
80 limit 79 to (english language and yr="2000 -Current")
81 40 not 80

Appendix S2: Inclusion and Exclusion Criteria

Inclusion Criteria: *all* of the following:

- Studies including children 1 month to 9 years, presenting with clinically diagnosed severe pneumonia (current or previous WHO definition, or other clinical criteria);
- Studies reporting on chest x-ray (CXR) *OR* lung ultrasound
- Studies from low-and middle-income countries for CXR, or any country for LUS
- Studies reporting any of the following outcomes, in relation to the role of CXR or LUS in children presenting with severe pneumonia:
    - Indications for imaging
    - Role of the imaging in diagnosis, where there is relevance to clinical decision making, including diagnostic accuracy of LUS
    - Role of the imaging in management
    - Impact of imaging findings on patient outcomes
    - Practical considerations relevant to low-and middle-income country settings (including feasibility and inter-reader or inter-operator reliability)
- Interventional studies, observational studies, systematic review articles and case series' with more than 20 participants.

Exclusion criteria: *any* of the following:

- Studies which exclude children with severe pneumonia
- Studies reporting imaging features of a specific aetiology of pneumonia
- Narrative reviews, letters, conference abstracts and opinion articles, case reports or small case series (< 20 patients)
- Studies not available in English

Appendix S3: Distribution of chest x-ray (CXR) studies

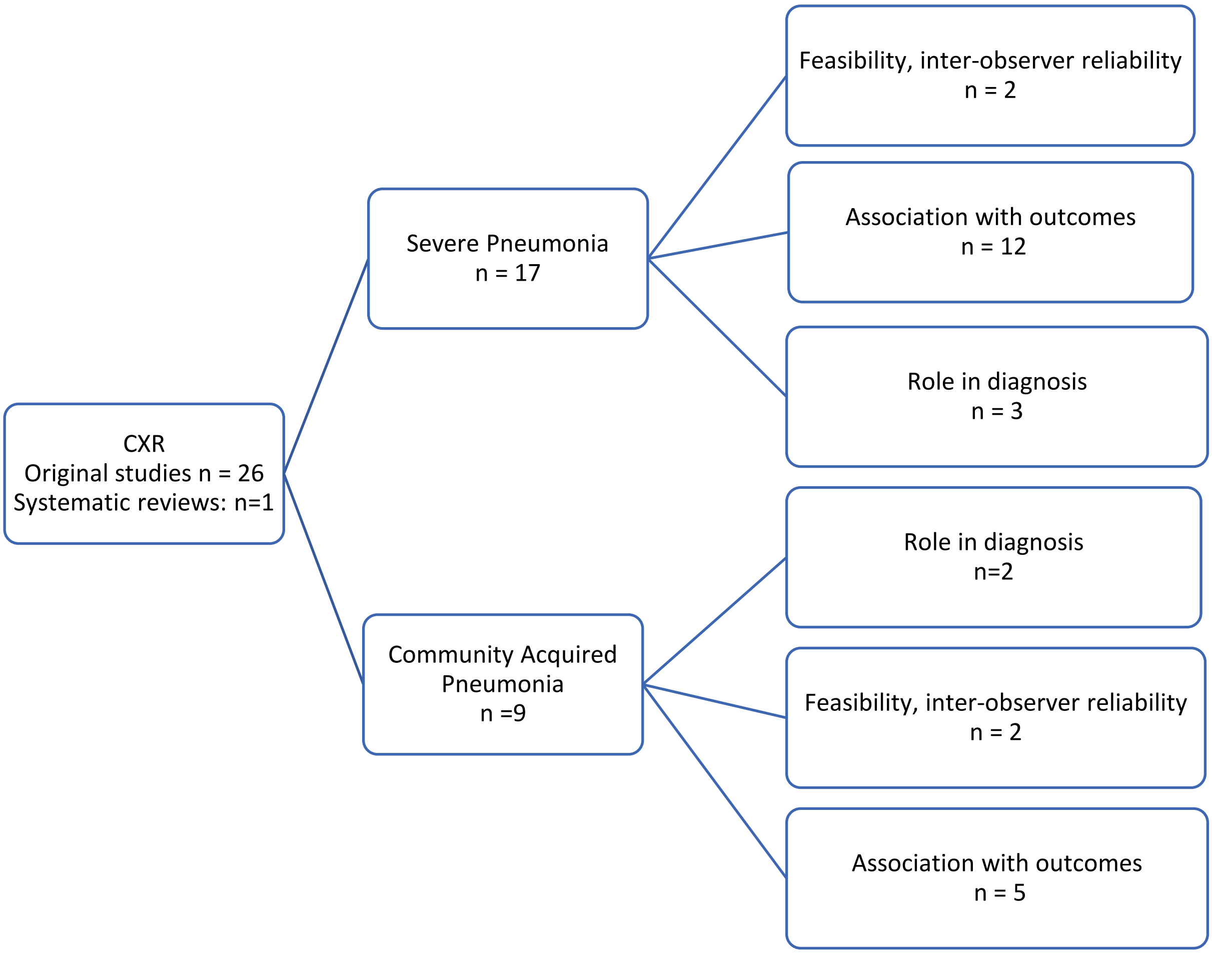

Appendix S4: Distribution of Lung Ultrasound (LUS) studies

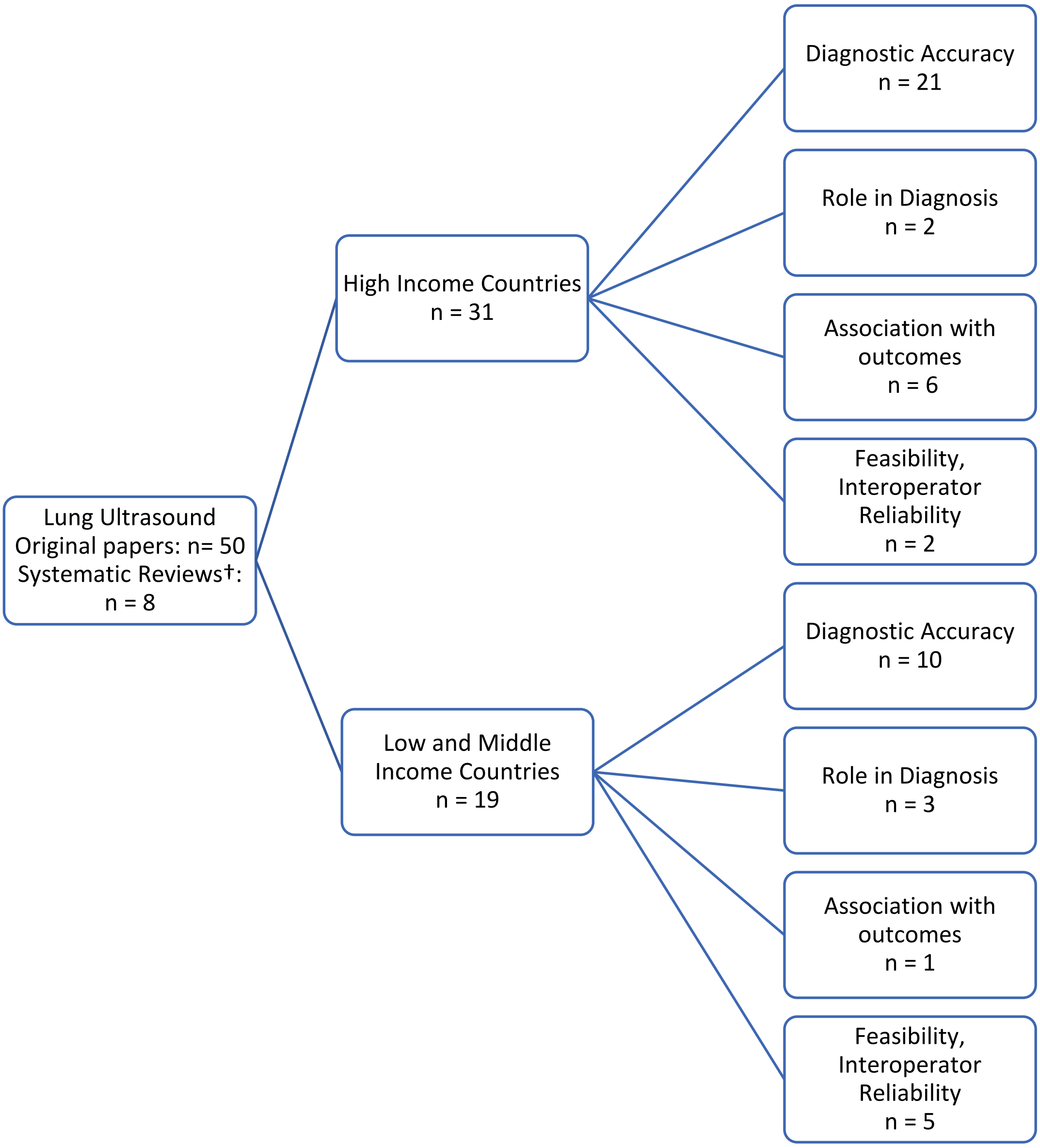

†All LUS systematic reviews looked at Diagnostic Accuracy as the primary outcome
